# Thermal Vibration of Thick FGM Conical Shells by Using Third-Order Shear Deformation Theory

**DOI:** 10.3390/ma17102403

**Published:** 2024-05-16

**Authors:** Chih-Chiang Hong

**Affiliations:** Department of Mechanical Engineering, Hsiuping University of Science and Technology, Taichung 412-406, Taiwan; cchong@mail.hust.edu.tw

**Keywords:** thick FGM, TSDT, thermal vibration, nonlinear coefficient

## Abstract

A time-dependent third-order shear deformation theory (TSDT) approach on the displacements of thick functionally graded material (FGM) conical shells under dynamic thermal vibration is studied. Dynamic equations of motion with TSDT for thick FGM conical shells are applied directly with the partial derivative of variable R*θ in the curve coordinates (*x*, θ, *z*) instead of *y* in the Cartesian coordinates (*x*, *y*, *z*) for thick FGM plates, where R* is the middle-surface radius at any point on conical shells. The generalized differential quadrature (GDQ) numerical method is used to solve the dynamic differential equations in equilibrium matrix forms under thermal loads. It is the novelty of the current study to identify the parametric effects of shear correction coefficient, environment temperature, TSDT model, and FGM power law index on the displacements and stresses in the thick conical shells only subjected to sinusoidal heating loads. The physical parts with values on the length-to-thickness ratio equals 5, and 10 FGMs can be used in an area of an airplane engine that usually operates near more than 1000 K of temperatures when the thermal stress is considered and affected. The important findings of the presented study are listed as follows. The values of normal stress are in decreasing tendencies with time in cases when the coefficient $c_{1}$ equals 0.925925/mm^2^ in TSDT and length-to-thickness ratio equals 5. The shear stress values in *x* plane *z* direction on the minor middle-surface radius (*r*) equals the major middle-surface radius (*R*) over 8 and length-to-thickness ratio equals to 5 can withstand *T* = 1000 K of pressure.

## 1. Introduction

The importance of the essential background study is to find the temperature effect of sinusoidal thermal loading on the displacement and stress. In the thick materials, the third-order shear deformation theory (TSDT) of displacements is used for more shear effect than the first-order shear deformation theory (FSDT) mode. The functionally graded material (FGM) can operate usually with less displacement and stress values under higher temperature, e.g., a 1000 K environment.

Many recent works in the literature are devoted to studying the mechanical behavior of conical shells made of functionally graded materials (FGMs). Soureshjani et al. [1] studied the numerical solutions of the generalized differential quadrature (GDQ) method for critical buckling pressure by using FSDT. Ansari et al. [2] used the von Karman geometric nonlinearity and higher-order shear deformation theory (HSDT) to study the numerical solutions of the variational differential quadrature (VDQ) method for maximum deflection. The VDQ method was a weak formulation form for governing equations and then was discretized directly by using time differential operators and the arc-length continuation scheme. Das et al. [3] used the modified Hertzian contact theory to study the time response of numerical results in the finite element method (FEM) for power law exponent materials Ti–6Al–4V-ZrO2. Shakouri [4] presented the Donnell’s FSDT to investigate the vibration behavior in the thermal environment. Sofiyev [5] presented a literature review in the realms of the vibration and buckling engineering of nuclear, space, marine, electronics, and biomedical. Taraghi et al. [6] used analytical models and experimental methods to study the buckling under external pressure. Yang et al. [7] used the FSDT to study the vibration results under aerodynamic load. Javed [8] investigated the free vibration by using TSDT and spline approximation. Mouli et al. [9] used the FSDT and commercial finite element tool ANSYS to study the free vibration. It would be a novelty of this study to consider both the two directional frequency of vibrations and nonlinear coefficient terms in the TSDT among the thermal vibration studies for thick FGM conical shells.

Some GDQ studies for thick FGM spherical shells, circular cylindrical shells, plates, and plates-cylindrical shells employed the parameters effect of TSDT, heating loads, and environment temperature. The investigations by using the TSDT model approach for thick material were more complicated than the FSDT model approach used for thin material. Hong [10] studied the GDQ solutions of thick plates by including the TSDT model. The material properties of power-law function for thick plates were used. The displacements and thermal stresses of thick plates were also presented. Hong [11] presented the GDQ results of thick circular cylindrical shells by using the TSDT model. The von Karman type of strain-displacement equations and partial derivatives of displacements with respect to thickness direction are assumed. Parametric effect studies of power law index and environment temperature on the circular cylindrical shells were presented.

In this study, the time responses of displacements and stresses are investigated by using the TSDT model and effect of computed shear coefficient in laminated conical shells with four simply supported edges. It is the objective of the current study to identify the parametric effects of TSDT model, environment temperature, and the power law index of FGMs on the displacements and stresses in the thick conical shells under heating loads. The aim of the study is to provide the GDQ results in the thermal vibration analysis for FGM thick conical shells.

## 2. Procedures of Formulations

**Figure 1:**
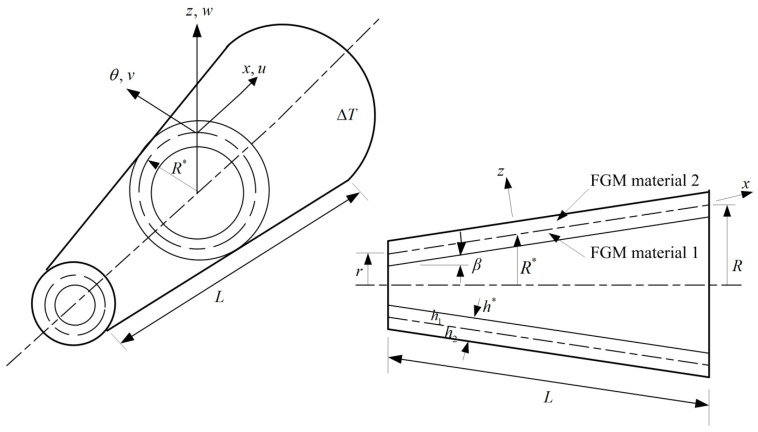
Two constituent materials-laminated thick FGM conical shells under thermal loads.

A point (*x*, θ, *z*) of curve coordinates in two constituent materials-laminated FGM conical shells under heating loads with temperature difference ∆T is shown in Figure 1 with the thickness $h_{1}$ of the inner layer constituent material 1 and thickness $h_{2}$ of the outer layer constituent material 2. *L* is the length of conical shells. $h^{*}$ is the total thickness of conical shells. *r* is the minor middle-surface radius at *x* = 0, and *R* is the major middle-surface radius at *x* = *L*. β is the semi-vertex angle. The power-law function with power law index $R_{n}$ is used and expressed in material properties ($E_{fgm}$, $\nu_{fgm}$) by Hong [11], as follows.

(1) $$E_{fgm}=\left(E_{2}-E_{1}\right)[\left(z+h^{*}/2\right)/h^{*}{]}^{R_{n}}+E_{1},$$

(2) $$\nu_{fgm}=(\nu_{1}+\nu_{2})/2,$$

in which $E_{2}$ and $E_{1}$ are Young’s modulus, $\nu_{2}$ and $\nu_{1}$ are Poisson’s ratios of the constituent material 2 and constituent material 1, respectively.

The individual constituent material properties (e.g., for $E_{2}$ and $E_{1}$) are used and expressed in functions of environment temperature (*T*) by Chi and Chung [12] as follows.

(3) $$P_{i}=P_{0}(P_{-1}T^{-1}+1+P_{1}T+P_{2}T^{2}+P_{3}T^{3}),$$

in which $P_{0}$, $P_{-1}$, $P_{1}$, $P_{2}$, and $P_{3}$ are coefficients of temperature. 

At any point (*x*, θ, *z*) of thick FGM conical shells, the time dependence of nonlinear displacements *u*, *v*, and w equations are applied to the coefficient $c_{1}$ term of TSDT by Lee et al. [13] with the parameter $R^{*}=r+x\sin\beta$ instead of the *R*, as follows:

(4) $$u=u_{0}\left(x,\theta ,t\right)+z\phi_{x}\left(x,\theta ,t\right){-c}_{1}z^{3}(\phi_{x}+\frac{\partial w}{\partial x}),$$

(5) $$v=v_{0}\left(x,\theta ,t\right)+z\phi_{\theta }\left(x,\theta ,t\right){-c}_{1}z^{3}(\phi_{\theta }+\frac{\partial w}{R^{*}\partial \theta }),$$

(6) $$w=w\left(x,\theta ,t\right),$$

where $u_{0}$, $v_{0}$, and w are the x, θ axes tangential and z axis transverse displacements, respectively, on the middle-plane of conical shells. $\phi_{x}$ and $\phi_{\theta }$ are the shear rotations. $R^{*}$ is the middle-surface radius of the conical shells at (*x*, θ, *z*). When the value β=0 is used, it becomes a case of circular cylindrical shell. *t* is the time, and $c_{1}=4/(3{h^{*}}^{2})$ expression is used for the TSDT model in nonlinear vs. $z^{3}$. When $c_{1}=0$ is used in Equations (4) and (5) for the thin material, then the displacement expression became the more simpler FSDT model in linear vs. z.

Normal stress denotations with $\sigma_{x}=\sigma_{xx}$, $\sigma_{\theta }=\sigma_{\theta \theta }$, and shear stress denotations with $\sigma_{x\theta }$, $\sigma_{\theta z}$, and $\sigma_{xz}$ on the (*k*)th layer of conical shells can be expressed in functions of stiffness, strains ($\varepsilon_{x}$, $\varepsilon_{\theta }$, $\varepsilon_{x\theta }$, $\varepsilon_{\theta z}$, $\varepsilon_{xz}$), and ∆T, as in Lee and Reddy [14] and Whitney [15]. The expression of ∆T between conical shells and curing area with linear and uncouple effects vs. *z* is given in the equation as follows:

(7) $$∆T=\frac{z}{h^{*}}T_{1}(x,\theta ,t),$$

in which $T_{1}$ is temperature parameter. For no heat generation, the equation of heat conduction in simplified form is applied by Hong [10] with the variable $R^{2}\partial \theta^{2}$ instead of the $\partial y^{2}$, as follows:

(8) $$K\left(\frac{\partial^{2}∆T}{\partial x^{2}}+\frac{\partial^{2}∆T}{R^{2}\partial \theta^{2}}+\frac{\partial^{2}∆T}{\partial z^{2}}\right)=\frac{\partial ∆T}{\partial t},$$

where $K=K_{fgm}/(\rho_{fgm}{C_{v}}_{fgm})$ in which $K_{fgm}$ is thermal conductivity, $\rho_{fgm}$ is density, and ${C_{v}}_{fgm}$ is specific heat. It can be reduced to the following equation to obtain the frequency γ of sinusoidal heat flux under heating loads:

(9) $$\frac{L^{2}}{K\pi^{2}[1+(L/R{)}^{2}]}\gamma \cos\left(\gamma t\right)+\sin\left(\gamma t\right)=0$$

For Equation (9), there is more detail to provide a step-by-step derivation in Appendix A.

Dynamic equations of motion for thick FGM conical shells with TSDT are applied by Hong [10] and by Reddy [16] with the variables $R^{*}\partial \theta$ and ${R^{*}}^{2}\partial \theta^{2}$ instead of the ∂y and $\partial y^{2}$, respectively, as follows:

(10) $$\frac{\partial N_{xx}}{\partial x}+\frac{1}{R^{*}}\frac{\partial N_{x\theta }}{\partial \theta }=I_{0}\frac{\partial^{2}u_{0}}{\partial t^{2}}+J_{1}\frac{\partial^{2}\phi_{x}}{\partial t^{2}}-c_{1}I_{3}\frac{\partial^{2}}{\partial t^{2}}\left(\frac{\partial w}{\partial x}\right),$$

(11) $$\frac{\partial N_{x\theta }}{\partial x}+\frac{1}{R^{*}}\frac{\partial N_{\theta \theta }}{\partial \theta }=I_{0}\frac{\partial^{2}v_{0}}{\partial t^{2}}+J_{1}\frac{\partial^{2}\phi_{\theta }}{\partial t^{2}}-c_{1}I_{3}\frac{\partial^{2}}{\partial t^{2}}(\frac{\partial w}{R^{*}\partial \theta }),$$

(12) $$\frac{\partial {\bar{Q}}_{x}}{\partial x}+\frac{1}{R^{*}}\frac{\partial {\bar{Q}}_{\theta }}{\partial \theta }+c_{1}\left(\frac{\partial^{2}P_{xx}}{\partial x^{2}}+\frac{2}{R^{*}}\frac{\partial^{2}P_{x\theta }}{\partial x\partial \theta }+\frac{1}{{R^{*}}^{2}}\frac{\partial^{2}P_{\theta \theta }}{\partial \theta^{2}}\right)+q=I_{0}\frac{\partial^{2}w}{\partial t^{2}}-{c_{1}}^{2}I_{6}\frac{\partial^{2}}{\partial t^{2}}(\frac{\partial^{2}w}{\partial x^{2}}+\frac{1}{{R^{*}}^{2}}\frac{\partial^{2}w}{\partial \theta^{2}})+c_{1}[I_{3}\frac{\partial^{2}}{\partial t^{2}}\left(\frac{\partial u_{0}}{\partial x}+\frac{1}{R^{*}}\frac{\partial v_{0}}{\partial \theta }\right)+J_{4}\frac{\partial^{2}}{\partial t^{2}}\left(\frac{\partial \phi_{x}}{\partial x}+\frac{1}{R^{*}}\frac{\partial \phi_{\theta }}{\partial \theta }\right)],$$

(13) $$\frac{\partial {\bar{M}}_{xx}}{\partial x}+\frac{1}{R^{*}}\frac{\partial {\bar{M}}_{x\theta }}{\partial \theta }-{\bar{Q}}_{x}=\frac{\partial^{2}}{\partial t^{2}}\left(J_{1}u_{0}+K_{2}\phi_{x}{-c}_{1}J_{4}\frac{\partial w}{\partial x}\right),$$

(14) $$\frac{\partial {\bar{M}}_{x\theta }}{\partial x}+\frac{1}{R^{*}}\frac{\partial {\bar{M}}_{\theta \theta }}{\partial \theta }-{\bar{Q}}_{\theta }=\frac{\partial^{2}}{\partial t^{2}}\left(J_{1}v_{0}+K_{2}\phi_{\theta }{-c}_{1}J_{4}\frac{1}{R^{*}}\frac{\partial w}{\partial \theta }\right),$$

where ${\bar{M}}_{\alpha \beta }=M_{\alpha \beta }-c_{1}P_{\alpha \beta }$, ${\bar{Q}}_{\alpha }=Q_{\alpha }-3c_{1}R_{\alpha }$, (α,β=x,θ),

$$\{\begin{array}{c} N_{xx}\\ N_{\theta \theta }\\ N_{x\theta } \end{array}\}=\int_{-\frac{h^{*}}{2}}^{\frac{h^{*}}{2}}\{\begin{array}{c} \sigma_{xx}\\ \sigma_{\theta \theta }\\ \sigma_{x\theta } \end{array}\}dz,\;\{\begin{array}{c} M_{xx}\\ M_{\theta \theta }\\ M_{x\theta } \end{array}\}=\int_{-\frac{h^{*}}{2}}^{\frac{h^{*}}{2}}\{\begin{array}{c} \sigma_{xx}\\ \sigma_{\theta \theta }\\ \sigma_{x\theta } \end{array}\}zdz,\;\{\begin{array}{c} P_{xx}\\ P_{\theta \theta }\\ P_{x\theta } \end{array}\}=\int_{-\frac{h^{*}}{2}}^{\frac{h^{*}}{2}}\{\begin{array}{c} \sigma_{xx}\\ \sigma_{\theta \theta }\\ \sigma_{x\theta } \end{array}\}z^{3}dz,$$

$$\{\begin{array}{c} R_{\theta }\\ R_{x} \end{array}\}=\int_{-\frac{h^{*}}{2}}^{\frac{h^{*}}{2}}\{\begin{array}{c} \sigma_{\theta z}\\ \sigma_{xz} \end{array}\}z^{2}dz,\;\{\begin{array}{c} Q_{\theta }\\ Q_{x} \end{array}\}=\int_{-\frac{h^{*}}{2}}^{\frac{h^{*}}{2}}\{\begin{array}{c} \sigma_{\theta z}\\ \sigma_{xz} \end{array}\}dz,\;I_{i}=\sum_{k=1}^{N^{*}}\int_{k}^{k+1}\rho^{\left(k\right)}z^{i}dz,\;\left(i=0,1,2,\cdots ,6\right),$$

where $N^{*}$ is total number of constituent layers and $\rho^{\left(k\right)}$ is the density of (*k*)th constituent ply. $J_{i}=I_{i}-c_{1}I_{i+2}$, (i=1,4) and $K_{2}=I_{2}-2c_{1}I_{4}+c_{1}{\;}^{2}I_{6}$.

Assuming $\frac{\partial v_{0}}{\partial z}=-\frac{v_{0}}{R^{*}}$, $\frac{\partial u_{0}}{\partial z}=-\frac{u_{0}}{R^{*}}$, $\frac{\partial w}{\partial z}=\frac{\partial \phi_{x}}{\partial z}=\frac{\partial \phi_{\theta }}{\partial z}=0$, terms of $\frac{1}{2}(\frac{\partial w}{\partial x}{)}^{2}$, $\frac{\partial w}{\partial x}\frac{\partial w}{R^{*}\partial \theta }$, $\frac{1}{2}(\frac{\partial w}{R^{*}\partial \theta }{)}^{2}$ are in constant value and can be applied by Hong [11] with the parameter $R^{*}$ instead of the R. Thus, dynamic differential equations with TSDT expressed in equilibrium matrix forms can be obtained for thick FGM conical shells under heating loads. Also, the varied values of shear correction coefficient $k_{\alpha }$ are used for the integrals of shear stiffness ${\bar{Q}}_{i^{*}j^{*}}$ in which subscripts $i^{*}$, $j^{*}$ = 4, 5. 

The GDQ numerical method can be used to solve the dynamic differential equations in equilibrium matrix forms. In the method history for differential quadrature (DQ) was issued by Bert et al. [17]. After then, the GDQ method was presented by Shu and Du [18]. Usually, the GDQ method approximated the function f(x,θ) derivatives at an arbitrary grid point $\left(x_{i},\theta_{j}\right)$ in which subscripts *i* = 1,2,…,*N* and *j* = 1,2,…,*M* for two-dimensional grid points *N* and *M*. The oscillations of time sinusoidal displacement with frequency $\omega_{mn}$, in which subscripts *m* and *n* are the mode shape numbers, shear rotations, environment temperature, and sinusoidal heat flux with frequency γ due to ∆T, are applied for the thermal vibrations by Hong [10]. And the simple vibration of ∆T for thermal loads can be given in the following sinusoidal expression:

(15) $$∆T=\frac{z}{h^{*}}{\bar{T}}_{1}\sin(\pi x/L)\sin(\pi \theta /R)\sin(\gamma t),$$

in which γ is calculated directly from Equation (9) and ${\bar{T}}_{1}$ is the temperature amplitude.

## 3. Numerical Results and Discussions

The Visual Studio 2015 Lahey–Fujitsu Fortran high level language can be used and implemented as the computational tool. The author has written the software program based on Lahey–Fujitsu Fortran to perform the simulation. Also, the current study with the method can be used from previously published work for the circular cylindrical shells by the same author Hong [11]. This current manuscript can be applied with the same methodology and materials to obtain the results for the thick conical shells by referring the previous publication works by the same author Hong [19]. The curve coordinates $x_{i}$ and $\theta_{j}$ for the conical shells are used as follows to calculate the GDQ solutions with constituent layers in FGMs under sinusoidal displacement vibration and heating loads only.

(16) $$x_{i}=0.5[1-\cos\left(\frac{i-1}{N-1}\pi \right)]L$$

(17) $$\theta_{j}=0.5[1-\cos\left(\frac{j-1}{M-1}\pi \right)]R$$

Usually, the linear $k_{\alpha }$ in varied values are functions of $h^{*}$, $R_{n}$, and *T* used in the preliminary study of thick FGM conical shells. The dynamic thermal vibration for thick FGM plates has been presented by the same author Hong [10]. Currently, it is very interesting and getting further study in the field of thick FGM conical shells. There are many material combinations for functionally graded (FG) mode. Usually, the FGM constituent material in general used metal material are SUS304, Ti–6Al–4V, Al, and Ni; ceramic material are Si_3_N_4_, ZrO_2_, and Al_2_O_3_. Also, the constituent material can be in the intelligent form for its particular function used, e.g., piezoelectric material BaTiO_3_ and PZT, magnetostrictive material CoFe_2_O_4_, and Terfenol-D. For the choosing that Si_3_N_4_/SUS304 used in the interesting present study, especially this type of composite materials possess temperature-dependent properties. The constituent material 1 on inner position is SUS304, and the FGM constituent material 2 on outer position is Si_3_N_4_ applied for the GDQ calculations. In thermal vibration of TSDT computation, the thermal loads in terms of ∆T in Equation (15) are only used for the external loads. Thermal vibration in the thermal loads are coupled with the temperature difference in frequency of sinusoidal heat flux Equation (9) and integrals of stiffness.

### 3.1. Dynamic Convergence

The iteration flow algorithm for the dynamic convergence studies is listed in Appendix B. The maximum value of displacement w can be founded particularly at center position $\left(x,\theta \right)=(L/2,2\pi /2)$. The center displacement w(L/2,2π/2)(mm) vs. N×M of dynamic convergence studies both in nonlinear TSDT with $c_{1}$ = 0.925925/mm^2^ and in linear with $c_{1}$ = 0/mm^2^ are investigated. For thick *L*/$h^{*}$ = 10, γ = 0.2618004/s and *L*/$h^{*}$ = 5, γ = 0.2618019/s under sinusoidal heat flux at *t* = 6 s, L/R=1, $h^{*}$ = 1.2 mm,$\;h_{1}$ = $h_{2}$ = 0.6 mm, *T* = 100 K, ${\bar{T}}_{1}$ = 1 K vs. *r* = 3*R*/4 are shown in Table 1. Also, the varied $k_{\alpha }$ and $\omega_{11}$ for $R_{n}$ values (0.5, 1, and 2) are considered in the sinusoidal oscillation. For example, in the *L*/$h^{*}$ = 5 of $c_{1}$ = 0.925925/mm^2^ case, when $R_{n}$ = 0.5, the values $k_{\alpha }$ = 0.111874 and $\omega_{11}$ = 0.001730/s are used; when $R_{n}$ = 1, the values $k_{\alpha }$ = 0.149001 and $\omega_{11}$ = 0.001730/s are used; when $R_{n}$ = 2, the values $k_{\alpha }$ = 0.231364 and $\omega_{11}$ = 0.001730/s are used. In the *L*/$h^{*}$ = 5 of $c_{1}$ = 0/mm^2^ case, when $R_{n}$ = 0.5, the values $k_{\alpha }$ = 0.111874 and $\omega_{11}$ = 0.004731/s are used; when $R_{n}$ = 1, the values $k_{\alpha }$ = 0.149001 and $\omega_{11}$ = 0.004191/s are used; when $R_{n}$ = 2, the values $k_{\alpha }$ = 0.231364 and $\omega_{11}$ = 0.003575/s are used. The 8.8 × 10^−5^ error accuracy can be found for nonlinear w(L/2,2π/2) of typical $c_{1}$ = 0.925925/mm^2^,$\;R_{n}$ = 2 and *L*/$h^{*}$ = 10 for N×M = 15 × 15 and 17 × 17 listed in Table 1. The 8.8 × 10^−5^ error accuracy can be considered acceptable in this presented preliminary study for conical shells. Table 1 also contains the comparison between the results obtained by using TSDT and FSDT ($c_{1}=0$). Consider Table 1, which reports the w(L/2,2π/2) after 6 s, e.g., at *t* = 12 s. Table 2 presents for the convergence of TSDT FGM conical shells with γ = 0.1309014/s, $\omega_{11}$ = 0.003457/s, *r* = 3*R*/4, and $R_{n}$ = 0.5 at *t* = 12 s. It can be shown that the smaller value of the displacement is decreasing as the number of grid points N×M increases. Thus, grid points N×M = 17 × 17 can be considered in a good convergence and used in the GDQ calculations of time responses for thick FGM conical shells. Also, the $\sigma_{x}$, $\sigma_{\theta }$, $\sigma_{x\theta }$, $\sigma_{\theta z}$, and $\sigma_{xz}$ on the FGMs can be calculated. There are no other researcher results in this area of research. Without additional evidence for the validation of the results, it would be premature to definitively claim sole authorship within this research area. The calculated similar values of concern cylindrical shells were studied by Brischetto and Torre [20], although there are some available similar but different analysis models used for the interesting cases.

### 3.2. Time Response

**Figure 2:**
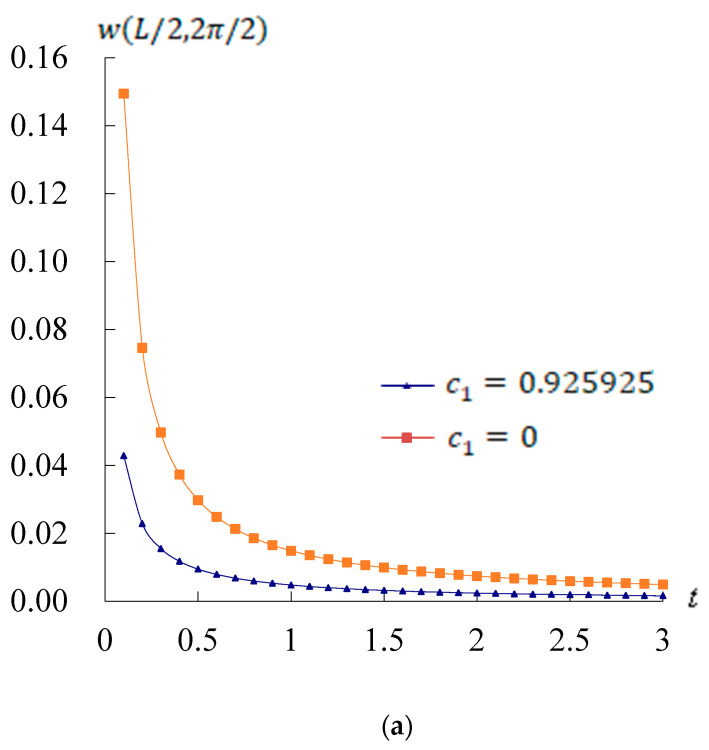

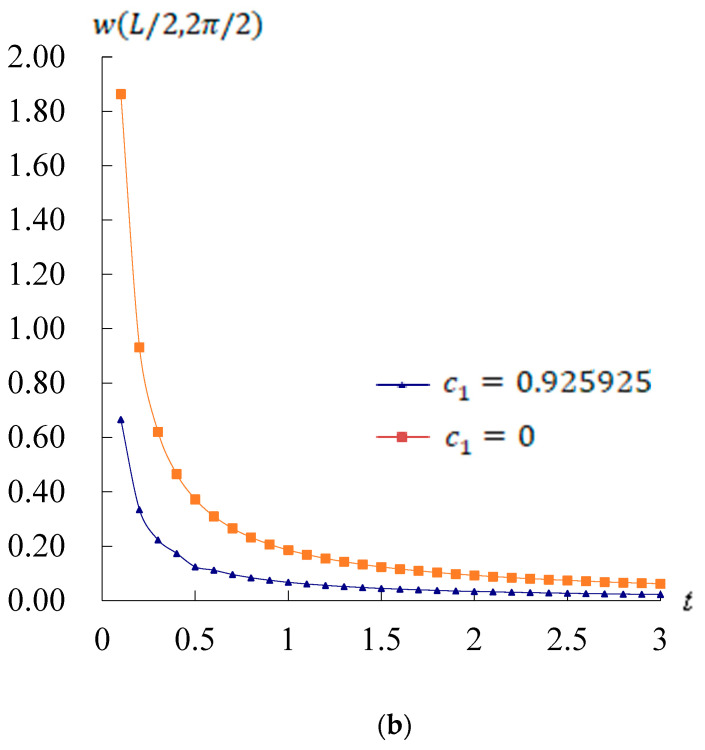
w(L/2,2π/2)(mm) vs. *t*(s) with *r* = 3*R*/4: (**a**) for *L*/$h^{*}$ = 5; (**b**) for *L*/$h^{*}$ = 10.

The time responses of w(L/2,2π/2) are computed with γ of sinusoidal heat flux and $\omega_{11}$ of sinusoidal displacement vibration. Figure 2 shows the responses of w(L/2,2π/2)(mm) versus *t*(s) for $c_{1}$ = 0.925925/mm^2^ and $c_{1}$ = 0/mm^2^, respectively, with *L*/$h^{*}$ = 5 and 10, L/R=1, $R_{n}=1$, $k_{\alpha }$ = 0.120708, *T* = 100 K, ${\bar{T}}_{1}$ = 1 K, and *r* = 3*R*/4. The w(L/2,2π/2) maximum value 0.149393 mm is found at *t* = 0.1 s for *L*/$h^{*}$ = 5 and $c_{1}$ = 0/mm^2^, as shown in Figure 2a. The w(L/2,2π/2) maximum value 1.863387 mm is found at *t* = 0.1 s for *L*/$h^{*}$ = 10 and $c_{1}$ = 0/mm^2^, as shown in Figure 2b. The w(L/2,2π/2) values are in decreasing tendencies for both $c_{1}$ = 0.925925/mm^2^, $c_{1}$ = 0/mm^2^, and *L*/$h^{*}$ = 5, *L*/$h^{*}$ = 10. The w(L/2,2π/2) values in $c_{1}$ = 0/mm^2^ case are greater than that in $c_{1}$ = 0.925925/mm^2^ case. The w(L/2,2π/2) result clearly state the improvement obtained from using this third order derivation TSDT case of $c_{1}$ = 0.925925/mm^2^, as opposed to the first or second order derivation case of $c_{1}$ = 0/mm^2^.

**Figure 3:**
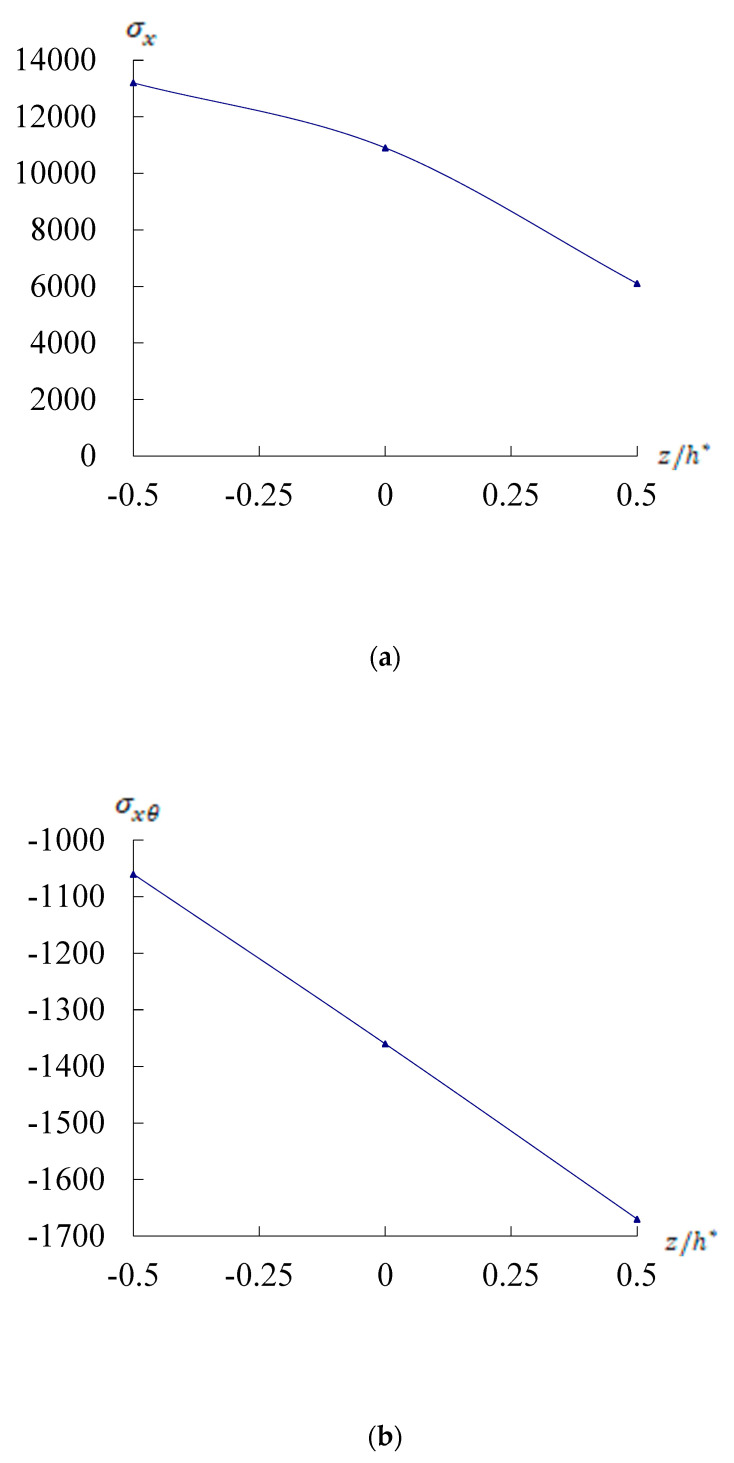

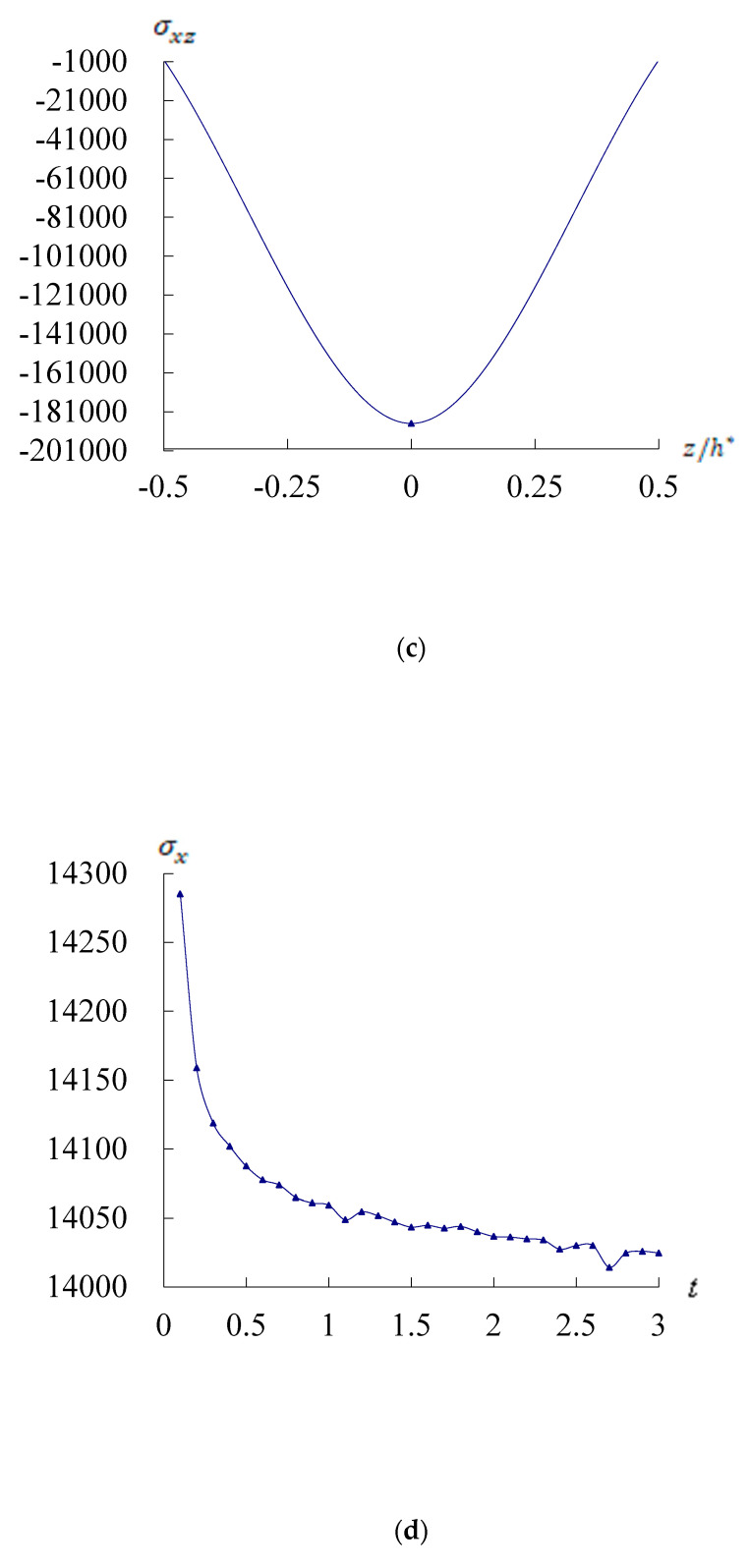

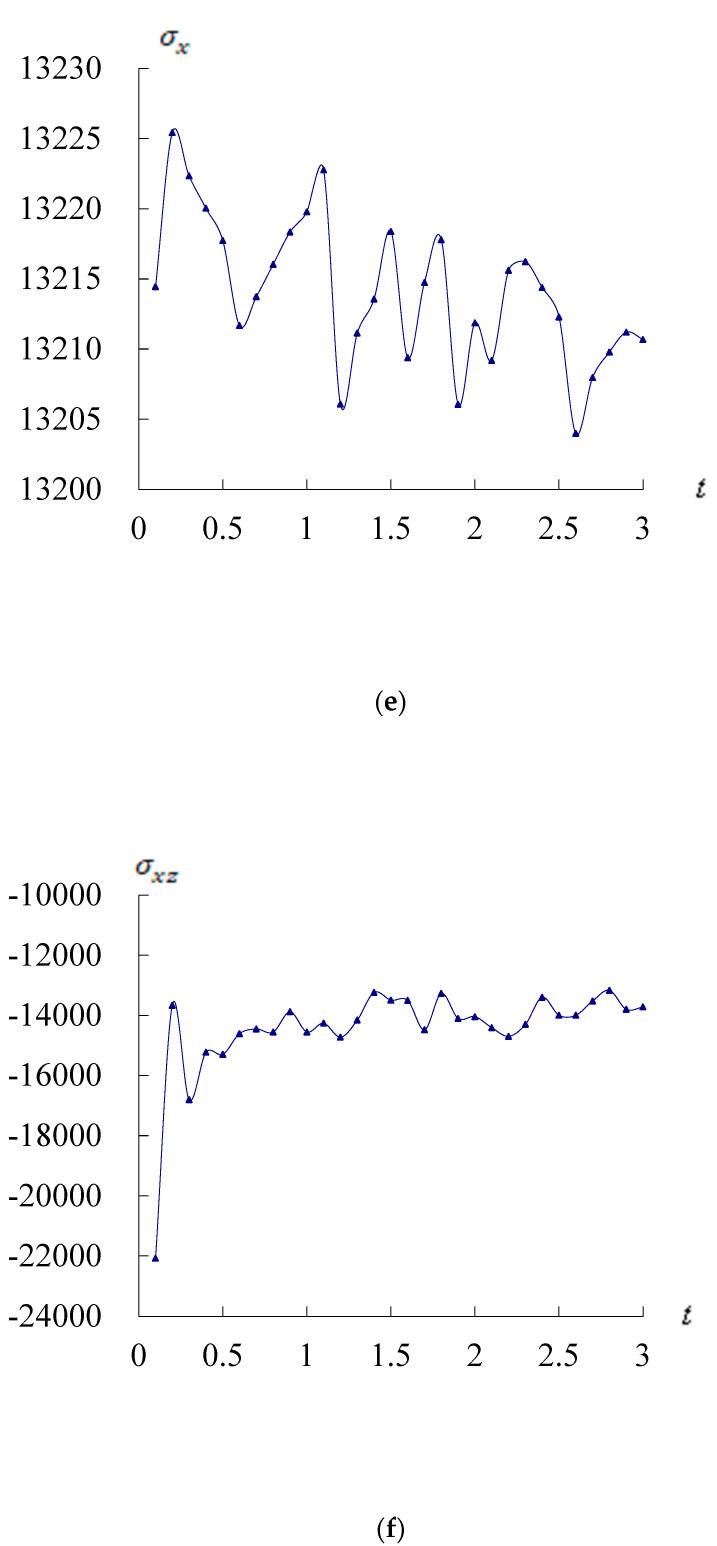

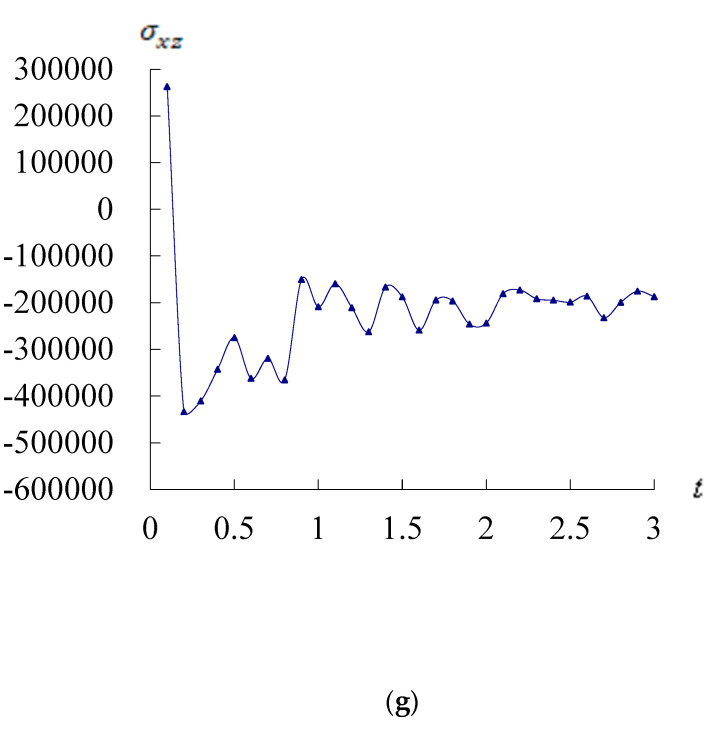
Stresses vs.$\;{z/h}^{*}$ and *t* with *r* = 3*R*/4: (**a**) $\sigma_{x}$(Pa) vs. ${z/h}^{*}$ for *L*/$h^{*}$ = 10; (**b**) $\sigma_{x\theta }$(Pa) vs. ${z/h}^{*}$ for *L*/$h^{*}$ = 10; (**c**) $\sigma_{xz}$(Pa) vs. ${z/h}^{*}$ for *L*/$h^{*}$ = 10; (**d**) $\sigma_{x}$(Pa) vs. *t*(s) for *L*/$h^{*}$ = 5; (**e**) $\sigma_{x}$(Pa) vs. *t*(s) for *L*/$h^{*}$ = 10; (**f**) $\sigma_{xz}$(Pa) vs. *t*(s) for *L*/$h^{*}$ = 5; (**g**) $\sigma_{xz}$(Pa) vs. *t*(s) for *L*/$h^{*}$ = 10.

Figure 3 shows the time responses of $\sigma_{x}$, $\sigma_{x\theta }$, and $\sigma_{xz}$ on *x* = *L*/2, θ=2π/2 for the conical shells. Figure 3a shows the $\sigma_{x}$(Pa) versus ${z/h}^{*}$. Figure 3b,c show the $\sigma_{x\theta }$ and $\sigma_{xz}$(Pa) versus ${z/h}^{*}$, respectively, at *t* = 3.0 s for ${a/h}^{*}$ = 10, $c_{1}$ = 0.925925/mm^2^, $R_{n}$ = 1, L/R=1, *T* = 100 K, ${\bar{T}}_{1}$ = 1 K, and *r* = 3*R*/4. The $\sigma_{xz}$ maximum value −1.8715 × 10^5^ Pa is found on *z* = 0.0$h^{*}$ for *L*/$h^{*}$ = 10. The $\sigma_{xz}$ absolute value for −1.8715 × 10^5^ Pa is found on *z* = 0.0$h^{*}$ and in greater value than $\sigma_{x}$ value 1.3210 × 10^4^ Pa on *z* = −0.5$h^{*}$. Figure 3d,e show the $\sigma_{x}$(Pa) time responses on inner surface *z* = −0.5$h^{*}$ for $R_{n}$ = 1,$\;c_{1}$ = 0.925925/mm^2^, *L*/$h^{*}$ = 5 and 10, respectively. The values of $\sigma_{x}$ are in decreasing tendencies for $c_{1}$ = 0.925925/mm^2^ and *L*/$h^{*}$ = 5. The $\sigma_{x}$ maximum value 1.3225 × 10^4^ Pa is found at *t* = 0.2 s and $\sigma_{x}$ values can be considered in order of 1.32 × 10^4^ Pa for *L*/$h^{*}$ = 10. Figure 3f,g show the time responses of the $\sigma_{xz}$(Pa) on middle surface *z* = 0.0$h^{*}$ for $R_{n}$ = 1,$\;c_{1}$ = 0.925925/mm^2^, *L*/$h^{*}$ = 5 and 10, respectively. The $\sigma_{xz}$ maximum absolute value for −4.3348 × 10^5^ Pa is found at *t* = 0.2 s for *L*/$h^{*}$ = 10.

### 3.3. Parameters Effect

**Figure 4:**
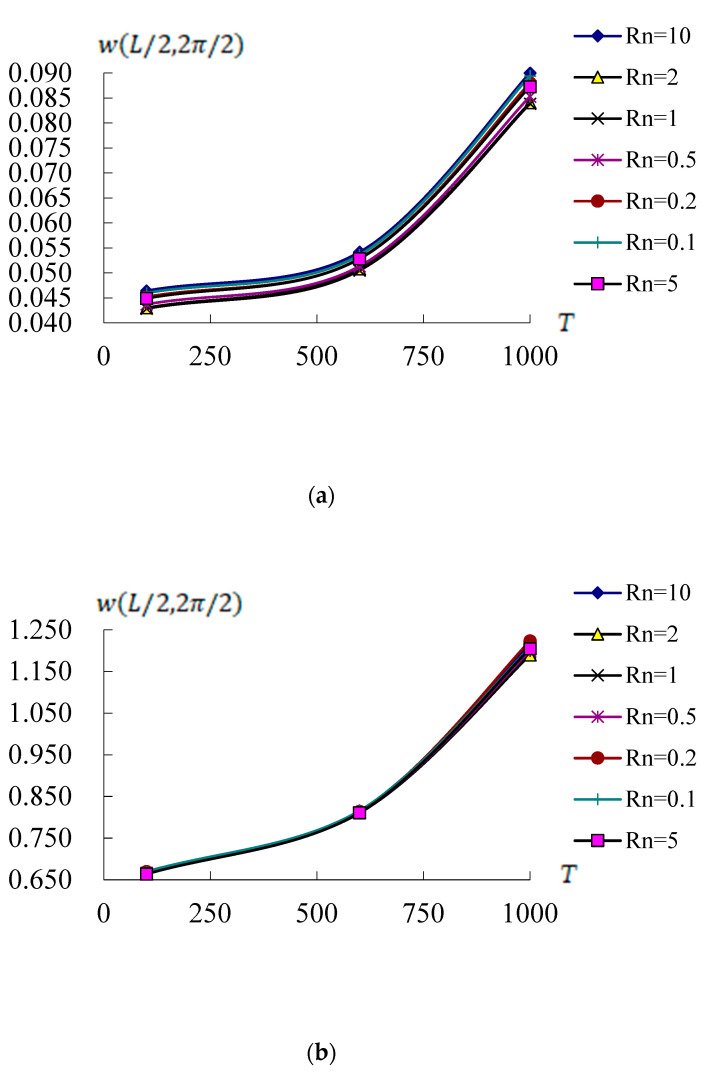
w(L/2,2π/2)(mm) versus *T*(K) with *r* = 3*R*/4: (**a**) for *L*/$h^{*}$ = 5 with $R_{n}$ from 0.1 to 10; (**b**) for *L*/$h^{*}$ = 10 with $R_{n}$ from 0.1 to 10.

Figure 4 shows the w(L/2,2π/2)(mm) response values vs. *T*(100 K, 600 K and 1000 K) and $R_{n}$(from 0.1 to 10) at *t* = 0.1 s under $c_{1}$ = 0.925925/mm^2^ for conical shells *L*/$h^{*}$ = 5 and 10, respectively, with L/R=1, ${\bar{T}}_{1}$ = 1 K and *r* = 3*R*/4. Figure 4a shows the w(L/2,2π/2) curves vs. *T* and $R_{n}$ for *L*/$h^{*}$ = 5 case, the w(L/2,2π/2) maximum value 0.089998 mm is found on *T* = 1000 K and $R_{n}$ = 10. The values of w(L/2,2π/2) are all in increasing tendencies for *T* from *T* = 100 K to *T* = 1000 K and all of $R_{n}$. The w(L/2,2π/2) amplitude for *L*/$h^{*}$ = 5 cannot withstand on *T* = 1000 K of environment. Figure 4b shows the w(L/2,2π/2) curves vs. *T* and $R_{n}$ for *L*/$h^{*}$ = 10 case, they are almost located in the same curves for all value of $R_{n}$. The w(L/2,2π/2) maximum value 1.223374 mm is found on *T* = 1000 K and $R_{n}$ = 0.2. The w(L/2,2π/2) values are all in increasing tendencies for *T* and all of $R_{n}$, the w(L/2,2π/2) amplitude for *L*/$h^{*}$ = 10 also cannot withstand on *T* = 1000 K of environment.

More interpretation for the stresses can be calculated from the following equations when the displacements and shear rotations are already computed. Thus, a peculiarly maximum values occur at *T* = 600 K for displacements and stresses.

(18) $$\begin{array}{l} \sigma_{x}=\{[\sum_{l=1}^{N}A_{i,l}^{\left(1\right)}U_{l,j}+\frac{z}{L}\sum_{l=1}^{N}A_{i,l}^{\left(1\right)}\varphi_{x}{{\;}_{\;}}_{l,j}-c_{1}z^{3}(\frac{1}{L}\sum_{l=1}^{N}A_{i,l}^{\left(1\right)}\varphi_{x}{{\;}_{\;}}_{l,j}+\frac{h^{*}}{L^{2}}\sum_{l=1}^{N}A_{i,l}^{\left(2\right)}W_{l,j})]\sin(\omega_{mn}t)\\ +\frac{1}{2}{\left(\frac{h^{*}L}{L^{2}}\sum_{l=1}^{N}A_{i,l}^{\left(1\right)}W_{l,j}\sin(\omega_{mn}t)\right)}^{2}-\alpha_{x}\frac{z}{h^{*}}\bar{T_{1}}\sin(\pi \;x_{i}/L)\sin(\pi \;\theta_{j}/R)\sin(\gamma t)\}{\bar{Q}}_{11}\\ +\{[\sum_{m=1}^{M}B_{j,m}^{\left(1\right)}V_{i,m}+\frac{z}{R}\sum_{m=1}^{M}B_{j,m}^{\left(1\right)}\varphi_{\theta }{{\;}_{\;}}_{i,m}-c_{1}z^{3}(\frac{1}{R}\sum_{m=1}^{M}B_{j,m}^{\left(1\right)}\varphi_{\theta }{{\;}_{\;}}_{i,m}+\frac{h^{*}}{L^{2}}\frac{L^{2}}{R^{2}}\sum_{m=1}^{M}B_{j,m}^{\left(2\right)}W_{i,m})]\sin(\omega_{mn}t)\\ +\frac{1}{2}{\left(\frac{h^{*}}{L^{2}}\frac{L^{2}}{R}\sum_{m=1}^{M}B_{j,m}^{\left(1\right)}W_{i,m}\sin(\omega_{mn}t)\right)}^{2}-\alpha_{\theta }\frac{z}{h^{*}}\bar{T_{1}}\sin(\pi \;x_{i}/L)\sin(\pi \;\theta_{j}/R)\sin(\gamma t)\}{\bar{Q}}_{12}\\ +\{[\frac{L}{R}\sum_{m=1}^{M}B_{j,m}^{\left(1\right)}U_{i,m}+\frac{z}{R}\sum_{m=1}^{M}B_{j,m}^{\left(1\right)}\varphi_{x}{\;}_{i,m}-c_{1}z^{3}(\frac{1}{R}\sum_{m=1}^{M}B_{j,m}^{\left(1\right)}\varphi_{x}{\;}_{i,m}+\frac{h^{*}}{L^{2}}\frac{L}{R}\sum_{l=1}^{N}\sum_{m=1}^{M}A_{i,l}^{\left(1\right)}B_{j,m}^{\left(1\right)}W_{l,m})\\ +\frac{R}{L}\sum_{l=1}^{N}A_{i,l}^{\left(1\right)}V_{l,j}+\frac{z}{L}\sum_{l=1}^{N}A_{i,l}^{\left(1\right)}\varphi_{\theta }{{\;}_{\;}}_{l,j}-c_{1}z^{3}(\frac{1}{L}\sum_{l=1}^{N}A_{i,l}^{\left(1\right)}\varphi_{\theta }{{\;}_{\;}}_{l,j}+\frac{h^{*}}{L^{2}}\frac{L}{R}\sum_{l=1}^{N}\sum_{m=1}^{M}A_{i,l}^{\left(1\right)}B_{j,m}^{\left(1\right)}W_{l,m})]\sin(\omega_{mn}t)\\ +(\frac{h^{*}L}{L^{2}}\sum_{l=1}^{N}A_{i,l}^{\left(1\right)}W_{l,j}\sin(\omega_{mn}t))(\frac{h^{*}}{L^{2}}\frac{L^{2}}{R}\sum_{m=1}^{M}B_{j,m}^{\left(1\right)}W_{l,m}\sin(\omega_{mn}t))\\ -\alpha_{x\theta }\frac{z}{h^{*}}\bar{T_{1}}\sin(\pi \;x_{i}/L)\sin(\pi \;\theta_{j}/R)\sin(\gamma t)\}{\bar{Q}}_{16} \end{array}$$

where $A_{i,l}^{\left(m\right)}$ and $B_{j,m}^{\left(m\right)}$ denote the weighting coefficients used in the GDQ calculations for the superscript (*m*)th-order derivative of functions, e.g., displacements ($U=u_{0}/L$, $V=v_{0}/R$ and $W=w/h^{*}$) and shear rotations ($\varphi_{x}(x,\theta )$ and $\varphi_{\theta }(x,\theta )$). $\alpha_{x}$ and $\alpha_{\theta }$ denoted the thermal expansion coefficients, $\alpha_{x\theta }$ denoted the thermal shear coefficient. And the stiffness ${\bar{Q}}_{ij}$ can be defined as follows:

(19) $${\bar{Q}}_{11}={\bar{Q}}_{22}=\frac{E_{fgm}}{1-\nu_{fgm}{\;}^{2}},{\bar{Q}}_{12}={\bar{Q}}_{21}=\frac{\nu_{fgm}E_{fgm}}{\left(1+z/R)(1-\nu_{fgm}{\;}^{2}\right)}\mathrm{and}{\bar{Q}}_{16}={\bar{Q}}_{26}={\bar{Q}}_{45}=0$$

**Figure 5:**
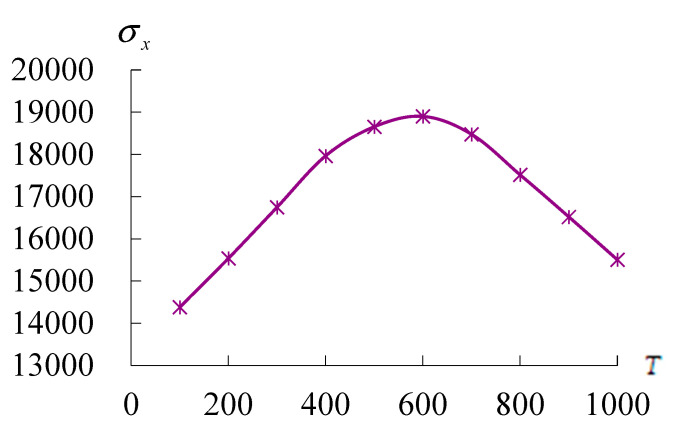
$\sigma_{x}$(Pa) versus *T*(K).

To provide physical justification for (18) used in the GDQ computation as follows, the values of stress along *x* direction ($\sigma_{x}$) are functions of symbolic parameters, e.g., displacements ($U_{l,j},\;V_{i,m},\;W_{l,j}$), shear rotations (${\phi_{x}}_{l,j},$ ${\phi_{\theta }}_{i,m}$), TSDT coefficient term ($c_{1}$), thermal loads temperature amplitude (${\bar{T}}_{1}$), environment temperature (*T*), sinusoidal heat flux frequency (γ), sinusoidal displacement fundamental frequency ($\omega_{11}$), located curve coordinates ($x_{i}$, $\theta_{j}$, z), and at time (*t*). One of the most important parameters is *T* to be considered for the stress $\sigma_{x}$ of FGM and used in higher temperature of environment. To provide the interpretation of the peculiar behavior of $\sigma_{x}$ on *T* = 600 K as follows, it is considered the increment 100 K used for *T* (100 K, 200 K, 300 K, 400 K, 500 K, 600 K, 700 K, 800 K, 900 K, and 1000 K) to calculate the values and interpret the behaviors of $\sigma_{x}$. Figure 5 shows the $\sigma_{x}$(Pa) on *x* = *L/2*, θ=2π/2 of inner surface *z* = −0.5$h^{*}$ vs. *T* and $R_{n}$ = 0.5 for conical shells *L*/$h^{*}$ = 5 with ${\bar{T}}_{1}$ = 1 K and *r* = 3*R*/4 at *t* = 0.1 s. Thus, the curve provided a maximum interpretation of the peculiar behavior of $\sigma_{x}$ around at *T* = 600 K. Then, the increment for *T* (100 K, 600 K, and 1000 K) is used to calculate the values and interpret the behaviors of $\sigma_{x}$.

**Figure 6:**
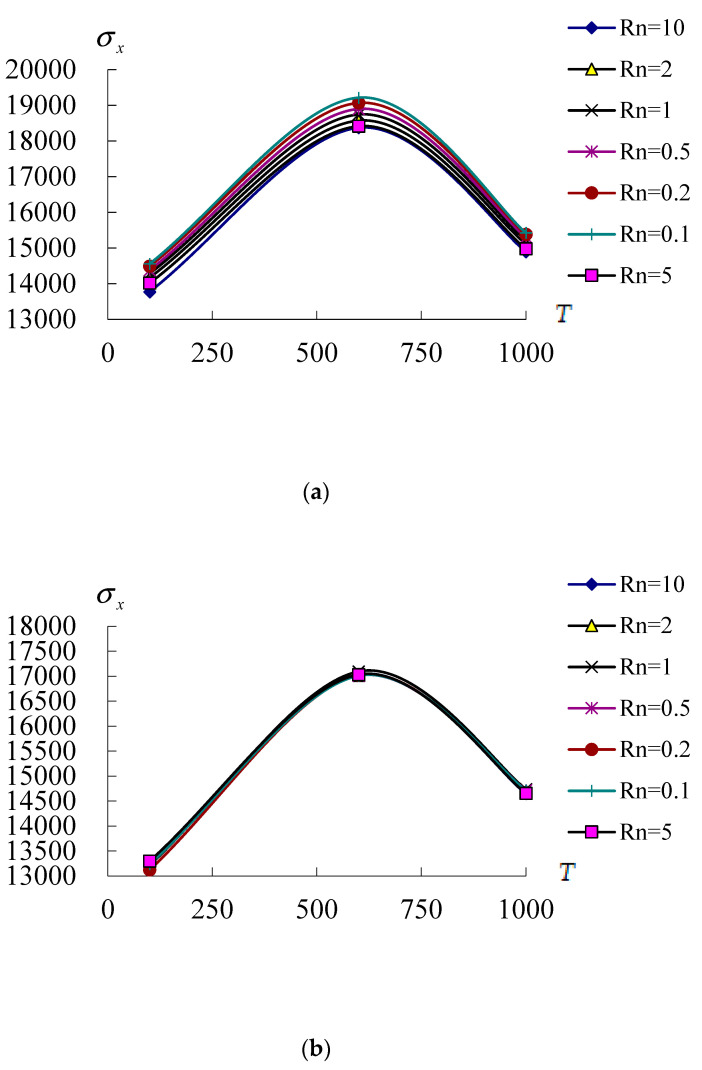
$\sigma_{x}$(Pa) versus *T*(K) with *r* = 3*R*/4: (**a**) for *L*/$h^{*}$ = 5; (**b**) for *L*/$h^{*}$ = 10.

Figure 6 shows the $\sigma_{x}$(Pa) on *x* = *L/2*, θ=2π/2 of inner surface *z* = −0.5$h^{*}$ vs. *T* (100 K, 600 K and 1000 K) and $R_{n}$(from 0.1 to 10) for conical shells *L*/$h^{*}$ = 5 and 10 with ${\bar{T}}_{1}$ = 1 K and *r* = 3*R*/4 at *t* = 0.1 s. Figure 6a shows the $\sigma_{x}$ curves vs. *T* and $R_{n}$ for *L*/$h^{*}$ = 5, the $\sigma_{x}$ values vs. *T* are all in increasing tendencies from *T* = 100 K to *T* = 600 K and then all in decreasing tendencies from *T* = 600 K to *T* = 1000 K for all of $R_{n}$. The $\sigma_{x}$ maximum value 1.9215 × 10^4^ Pa is found on *T* = 600 K and $R_{n}$ = 0.1. The $\sigma_{x}$ values of *L*/$h^{*}$ = 5 can withstand on *T* = 1000 K of environment. Figure 6b shows the $\sigma_{x}$ curves vs. *T* and $R_{n}$ for *L*/$h^{*}$ = 10, they are almost located in the same curves for all value of $R_{n}$. The $\sigma_{x}$ values vs. *T* are all in increasing tendencies from *T* = 100 K to *T* = 600 K, and then all in decreasing tendencies from *T* = 600 K to *T* = 1000 K for all of $R_{n}$. The $\sigma_{x}$ maximum value 1.7094 × 10^4^ Pa is found on *T* = 600 K. The $\sigma_{x}$ values of *L*/$h^{*}$ = 10 also can withstand on *T* = 1000 K of environment. Usually, the thermal stress occurred and be stood on the FGMs under thermal loading of extreme high temperatures by Noda [21]. The physical parts on values *L*/$h^{*}$ = 5 and 10 of FGMs can be used in area of airplane engine that usually operate near more than 1000 K of temperatures when the thermal stress to be considered and affected. To understand the effect of thermal loading on the thermal stress, the middle-near 600 K of temperatures can be used to study.

### 3.4. Compared Results

**Figure 7:**
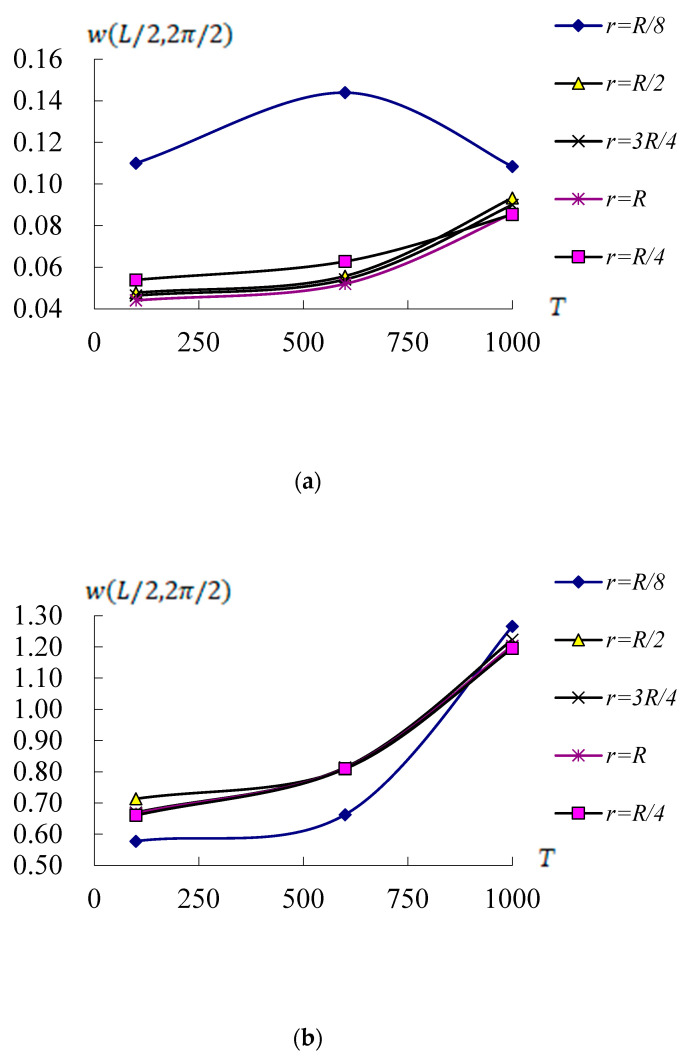
Compared w(L/2,2π/2)(mm) vs. *T*(K) and *r*: (**a**) for *L*/$h^{*}$ = 5 with $R_{n}$ = 10; (**b**) for *L*/$h^{*}$ = 10 with $R_{n}$ = 0.2.

Figure 7 shows the compared values of w(L/2,2π/2)(mm) vs. *T*(100 K, 600 K and 1000 K) for *r* (*R*/8, *R*/4, *R*/2, 3*R*/4 and *R*) of conical shells with $c_{1}$ = 0.925925/mm^2^, L/R=1 and ${\bar{T}}_{1}$ = 1 K at *t* = 0.1 s. Figure 7a shows the w(L/2,2π/2) values vs. *T* and *r* for *L*/$h^{*}$ = 5 and $R_{n}$ = 10. The w(L/2,2π/2) maximum value 0.143956 mm is found on *r* = *R*/8 and *T* = 600 K. The w(L/2,2π/2) values vs. *T* are in increasing tendencies from *T* = 100 K to *T* = 600 K and then in decreasing tendencies from *T* = 600 K to *T* = 1000 K for the case *r* = *R*/8. The w(L/2,2π/2) values for the case *r* = *R*/8 and *L*/$h^{*}$ = 5 can withstand on *T* = 1000 K of environment. Thus, physical parts on values $R_{n}$ = 10, *r* = *R*/8 and *L*/$h^{*}$ = 5 of FGMs can be used in an area of an airplane engine that usually operates near more than 1000 K of temperatures. To understand the effect of thermal loading on the displacement, the middle-near 600 K of temperatures can be used to study. Figure 7b shows the w(L/2,2π/2) values vs. *T* and *r* for *L*/$h^{*}$ = 10 and $R_{n}$ = 0.2. The w(L/2,2π/2) maximum value 1.265982 mm is found on *r* = *R*/8 and *T* = 1000 K. The w(L/2,2π/2) values are all in increasing tendencies vs. *T* for all values of *r.* The w(L/2,2π/2) values for *L*/$h^{*}$ = 10 cannot withstand on *T* = 1000 K of environment.

**Figure 8:**
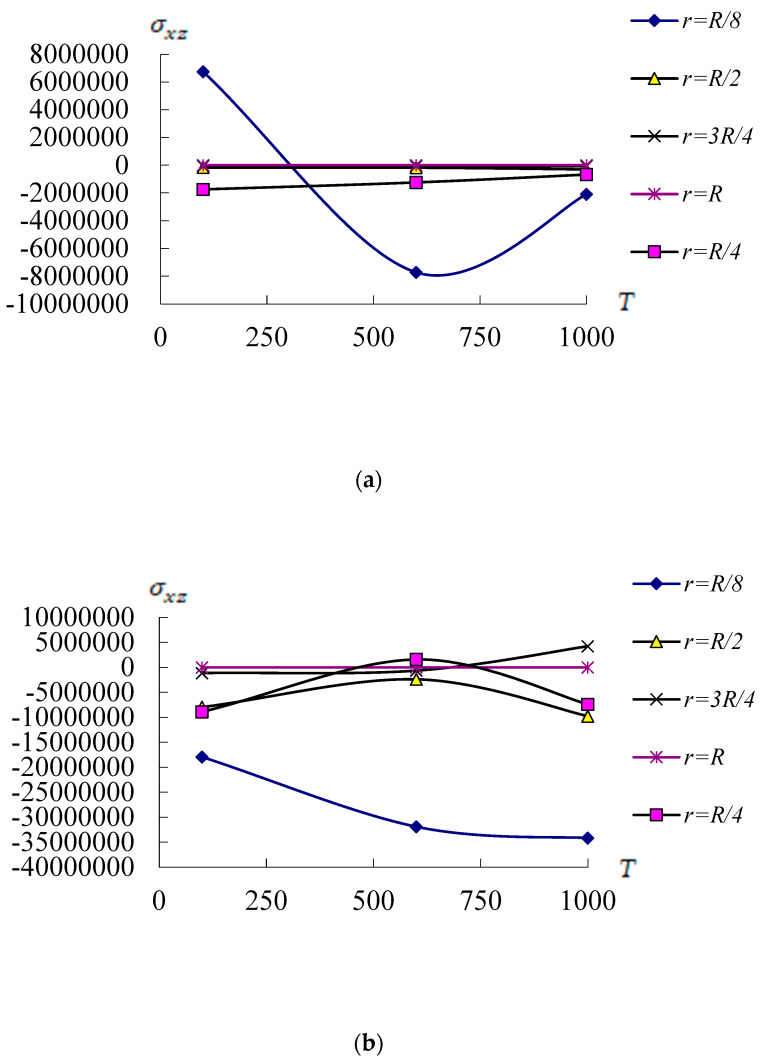
Compared $\sigma_{xz}$(Pa) vs. *T*(K) and *r*: (**a**) for *L*/$h^{*}$ = 5 with $R_{n}$ = 10; (**b**) for *L*/$h^{*}$ = 10 with $R_{n}$ = 0.2.

Figure 8 shows the compared values of $\sigma_{xz}$(Pa) on center position of middle surface *z* = 0.0$h^{*}$ vs. *T*(100 K, 600 K, and 1000 K) for *r*(*R*/8, *R*/4, *R*/2, 3*R*/4, and *R*) with $c_{1}$ = 0.925925/mm^2^. Figure 8a shows the values of $\sigma_{xz}$ vs. *T* and *r* in the nonlinear TSDT FGM conical shells for *L*/$h^{*}$ = 5, $R_{n}$ = 10, L/R=1, and ${\bar{T}}_{1}$ = 1 K at *t* = 0.1 s. The $\sigma_{xz}$ maximum value −7.7207 × 10^6^ Pa is found on *r* = *R*/8, *T* = 600 K and *L*/$h^{*}$ = 5. The $\sigma_{xz}$ values vs. *T* are in increasing tendencies from *T* = 300 K to *T* = 600 K and then in decreasing tendencies from *T* = 600 K to *T* = 1000 K for the case *r* = *R*/8. The $\sigma_{xz}$ values for the case *r* = *R*/8 and *L*/$h^{*}$ = 5 can withstand on *T* = 1000 K of environment. Figure 8b shows the $\sigma_{xz}$ values vs. *T* and *r* in the nonlinear TSDT FGM conical shells for *L*/$h^{*}$ = 10, $R_{n}$ = 0.2, L/R=1 and ${\bar{T}}_{1}$ = 1 K at *t* = 0.1 s. The $\sigma_{xz}$ maximum value −3.4165 × 10^7^ Pa is found on *r* = *R*/8, *T* = 1000 K and *L*/$h^{*}$ = 10. The $\sigma_{xz}$ values are all in increasing tendencies vs. *T* from *T* = 600 K to *T* = 1000 K for all values of *r.* The $\sigma_{xz}$ values of the *L*/$h^{*}$ = 10 cannot withstand on *T* = 1000 K of environment.

### 3.5. Limitations

For further study, the imperfection of FGMs can be considered. Also, the more details of imperfection in the material composition of FGMs can be referred to in the literature by Malikan and Eremeyev [22]. Also, the inhomogeneousness of FGMs can be studied in the future. The more details of inhomogeneousness in the material composition of FGMs can be referred in the literature by Dastjerdi and Akgöz [23].

## 4. Conclusions

Dynamic GDQ thermal vibration results are studied for the displacements and stresses in the of laminated thick FGM conical shells by considering the effects of computed shear coefficient and $c_{1}$ term of TSDT. The displacement values in $c_{1}$ = 0/mm^2^ case are greater than that $c_{1}$ = 0.925925/mm^2^ case for *L*/$h^{*}$ = 5 and *L*/$h^{*}$ = 10, respectively. It is over estimated in the center displacement values for the linear case. The values of stress $\sigma_{x}$ are in decreasing tendencies with time in $c_{1}$ = 0.925925/mm^2^ and *L*/$h^{*}$ = 5. The $\sigma_{xz}$ values on *r* = *R*/8 and *L*/$h^{*}$ = 5 can withstand *T* = 1000 K of the environment. Main highlights included: (a) thermal vibration of conical shells by TSDT are computed (b) with varied shear correction to obtain the displacements and stresses results; (c) FGM power law and environment temperature are considered. Main findings included: (d) result improvement obtained from using the TSDT case better than that FSDT case; and (e) compared values of displacement and stress versus environment temperature (100 K, 600 K, and 1000 K) for minor middle-surface radius (*R*/8, *R*/4, *R*/2, 3*R*/4, and *R*). A limitation would be that too many grid points used in the calculation would cause too much memory space occupied in RAM. Areas of future research in advanced thermal vibration of FGM conical shells would include studies of TDST and the nonlinear varied shear coefficient.

## Figures and Tables

**Table 1:** Convergence of TSDT FGM conical shells with *r* = 3*R*/4.

$c_{1}$(1/mm^2^)	$L/h^{*}$	GDQ Method	$\mathrm{Displacement}\;w\left(L/2,2\pi /2\right)$(mm) at *t* = 6 s		
		N×M	$R_{n}$ = 0.5	$R_{n}$ = 1	$R_{n}$ = 2
0.925925	10	7 × 7	0.011270	0.011308	0.011308
		9 × 9	0.011274	0.011275	0.011276
		11 × 11	0.011274	0.011275	0.011276
		13 × 13	0.011272	0.011272	0.011274
		15 × 15	0.011270	0.011272	0.011273
		17 × 17	0.011263	0.011267	0.011272
	5	7 × 7	0.000852	0.000851	0.000851
		9 × 9	0.000846	0.000846	0.000847
		11 × 11	0.000845	0.000846	0.000845
		13 × 13	0.000836	0.000838	0.000839
		15 × 15	0.000834	0.000836	0.000837
		17 × 17	0.000816	0.000822	0.000825
0	10	7 × 7	−0.045997	−0.044647	−0.077112
		9 × 9	0.028134	0.030911	0.034508
		11 × 11	0.028229	0.030891	0.034972
		13 × 13	0.028143	0.030926	0.034519
		15 × 15	0.028710	0.030805	0.034919
		17 × 17	0.028140	0.030926	0.034534
	5	7 × 7	0.002116	0.002409	0.002833
		9 × 9	0.002199	0.002478	0.002898
		11 × 11	0.002167	0.002443	0.002858
		13 × 13	0.002201	0.002480	0.002900
		15 × 15	0.002183	0.002461	0.002877
		17 × 17	0.002201	0.002480	0.002739

**Table 2:** Convergence of TSDT FGM conical shells with *r* = 3*R*/4 at *t* = 12 s.

$c_{1}$(1/mm^2^)	$L/h^{*}$	N×M	$w\left(L/2,2\pi /2\right)$$(\mathrm{mm})\;\mathrm{for}\;R_{n}$ = 0.5
0.925925	5	7 × 7	0.000071
		9 × 9	0.000064
		11 × 11	0.000063
		13 × 13	0.000056
		15 × 15	0.000053
		17 × 17	0.000050

## Data Availability

In the manuscript completely mentioned the data used to generate the figures and tables. Data are all available on request. The authors declare that all the data are generated by the author, and also that data are openly available.

## Appendix A

For no heat generation, the heat conduction equation can be given as follows:

(A1) $$K\left(\frac{\partial^{2}∆T}{\partial x^{2}}+\frac{\partial^{2}∆T}{R^{2}\partial \theta^{2}}+\frac{\partial^{2}∆T}{\partial z^{2}}\right)=\frac{\partial ∆T}{\partial t}.$$

And the sinusoidal expression ∆T can be given simply with linear vs. z, as follows:

(A2) $$∆T=\frac{z}{h^{*}}{\bar{T}}_{1}\sin(\pi x/L)\sin(\pi \theta )\sin(\gamma t),$$

then (A1) can be obtained as follows:

(A3) $$K\left(-\frac{z}{h^{*}}\frac{\pi^{2}}{L^{2}}{\bar{T}}_{1}\sin\left(\frac{\pi x}{L}\right)\sin\left(\pi \theta \right)\sin\left(\gamma t\right)-\frac{1}{R^{2}}\frac{z}{h^{*}}\pi^{2}{\bar{T}}_{1}\sin\left(\frac{\pi x}{L}\right)\sin\left(\pi \theta \right)\sin\left(\gamma t\right)\right)=\gamma \frac{z}{h^{*}}{\bar{T}}_{1}\sin\left(\frac{\pi x}{L}\right)\sin\left(\pi \theta \right)\cos\left(\gamma t\right),$$

thus:

(A4) $$K\left(-\frac{\pi^{2}}{L^{2}}-\frac{\pi^{2}}{R^{2}}\right)\sin\left(\gamma t\right)=\gamma \cos\left(\gamma t\right),$$

also:

(A5) $$-\frac{K\pi^{2}}{L^{2}}(1+\frac{L^{2}}{R^{2}})\sin\left(\gamma t\right)=\gamma \cos\left(\gamma t\right).$$

It can be reduced to the following equation:

(A6) $$\frac{L^{2}}{K\pi^{2}[1+(L/R{)}^{2}]}\gamma \cos\left(\gamma t\right)+\sin\left(\gamma t\right)=0.$$

## Appendix B

The five-degree polynomial equation can be obtained for the sinusoidal form of free vibration in dynamic equilibrium differential equations, as follows;

(A7) $$A(1)\lambda_{mn}{\;}^{5}+A(2)\lambda_{mn}{\;}^{4}+A(3)\lambda_{mn}{\;}^{3}+A(4)\lambda_{mn}{\;}^{2}+A(5)\lambda_{mn}+A(6)=0,$$

where

(A8) $$\lambda_{mn}=I_{0}\omega_{mn}{\;}^{2},$$

(A9) A(1)=−sd,

(A10) $$A(2)=(FH_{11}+FH_{22})sd+sc,$$

(A11) $$A(3)=-[(FH_{11}FH_{22}-FH_{12}FH_{12})sd+(FH_{11}+FH_{12})sc+sb],$$

(A12) $$A(4)=(FH_{11}FH_{22}-FH_{12}FH_{12})sc+(FH_{11}+FH_{22})sb+sa,$$

(A13) $$A(5)=-[(FH_{11}FH_{22}-FH_{12}FH_{12})sb+(FH_{11}+FH_{22})sa],$$

(A14) $$A(6)=(FH_{11}FH_{22}-FH_{12}FH_{12})sa,$$

in which $\omega_{mn}$ denotes the vibration frequency with mode shape numbers *m* and *n* in the subscripts,

(A15) $$sd={\left(K_{2}/I_{0}\right)}^{2},$$

(A16) $$sc=FH_{33}sd+FH_{44}K_{2}/I_{0},$$

(A17) $$sb=(FH_{33}FH_{55}+FH_{44}FH_{55}+FH_{33}FH_{44}-FH_{35}FH_{35}-FH_{34}FH_{34})K_{2}/I_{0}-FH_{45}FH_{45},$$

(A18) $$sa=FH_{33}FH_{44}FH_{55}+FH_{44}FH_{34}FH_{35}+FH_{35}FH_{34}FH_{45}-FH_{35}FH_{35}FH_{44}-FH_{34}FH_{34}FH_{55}-FH_{45}FH_{45}FH_{33},$$

and

(A19) $${FH}_{11}=A_{11}{\left(m\pi /L\right)}^{2}{+A}_{66}{\left(n\pi /R\right)}^{2},$$

(A20) $${FH}_{12}=(A_{12}{+A}_{66})(m\pi /L)(n\pi /R),$$

(A21) $${FH}_{22}=A_{66}{\left(m\pi /L\right)}^{2}{+A}_{22}{\left(n\pi /R\right)}^{2},$$

(A22) $${FH}_{33}=A_{55}{\left(m\pi /L\right)}^{2}{+A}_{44}{\left(n\pi /R\right)}^{2}{{+c}_{1}}^{2}H_{11}{\left(m\pi /L\right)}^{4}+{{(2c}_{1}}^{2}H_{12}+{{4c}_{1}}^{2}H_{66}{)(m\pi /L)}^{2}{\left(n\pi /R\right)}^{2}\;{+c_{1}}^{2}H_{22}{\left(n\pi /R\right)}^{4}-{3c}_{1}(2D_{55}-{3c}_{1}F_{55}{)(m\pi /L)}^{2}{-{3c}_{1}(2D_{44}-{3c}_{1}F_{44})(n\pi /R)}^{2},$$

(A23) $${FH}_{34}=A_{55}{m\pi /L{-(c}_{1}F}_{11}{{-c}_{1}}^{2}H_{11})\;{\left(m\pi /L\right)}^{3}{-(2c_{1}F}_{66}{{-2c}_{1}}^{2}H_{66}+{c_{1}F}_{12}{{-c}_{1}}^{2}H_{12})(m\pi /L){\left(n\pi /R\right)}^{2}{-(6c_{1}D}_{55}{{-9c}_{1}}^{2}F_{55})(m\pi /L),$$

(A24) $$FH_{35}=A_{44}n\pi /R-(c_{1}^{\;}F_{22}-c_{1}^{2}H_{22}){\left(n\pi /R\right)}^{3}-(2c_{1}^{\;}F_{66}-2c_{1}^{2}H_{66}+c_{1}^{\;}F_{12}-c_{1}^{2}H_{12}){\left(m\pi /L\right)}^{2}(n\pi /R)-(6c_{1}^{\;}D_{44}-9c_{1}^{2}F_{44})(n\pi /R),$$

(A25) $$FH_{44}=(D_{11}-2c_{1}F_{11}+c_{1}^{2}H_{11}){\left(m\pi /L\right)}^{2}+(D_{66}-2c_{1}F_{66}+c_{1}^{2}H_{66}){\left(n\pi /R\right)}^{2}+A_{55}-6c_{1}D_{55}+9c_{1}^{2}F_{55},$$

(A26) $$FH_{45}=(D_{12}+D_{66}-2c_{1}F_{12}+c_{1}^{2}H_{12}-2c_{1}F_{66}+c_{1}^{2}H_{66})(m\pi /L)(n\pi /R),$$

(A27) $$FH_{55}=(D_{66}-2c_{1}F_{66}+c_{1}^{2}H_{66}){\left(m\pi /L\right)}^{2}+(D_{22}-2c_{1}F_{22}+c_{1}^{2}H_{22}){\left(n\pi /R\right)}^{2}+A_{44}-6c_{1}D_{44}+9c_{1}^{2}F_{44},$$

(A28) $$(A_{i^{s}j^{s}},B_{i^{s}j^{s}},D_{i^{s}j^{s}},E_{i^{s}j^{s}},F_{i^{s}j^{s}},H_{i^{s}j^{s}})=\int_{\frac{-h^{*}}{2}}^{\frac{h^{*}}{2}}{\bar{Q}}_{i^{s}j^{s}}(1,z,z^{2},z^{3},z^{4},z^{6})dz,\;\left(i^{s},j^{s}=1,2,6\right),$$

(A29) $$(A_{i^{*}j^{*}},B_{i^{*}j^{*}},D_{i^{*}j^{*}},E_{i^{*}j^{*}},F_{i^{*}j^{*}},H_{i^{*}j^{*}})=\int_{\frac{-h^{*}}{2}}^{\frac{h^{*}}{2}}k_{\alpha }{\bar{Q}}_{i^{*}j^{*}}^{\;}(1,z,z^{2},z^{3},z^{4},z^{5})dz,\;(i^{*},j^{*}=4,5),$$

Then, the iteration flow algorithm of Newton’s method [24] can be used to find the real zeros $\lambda_{mn}$ of polynomial Equation (A7). Firstly, a first gauss of $\lambda_{mn}$ is chosen and read in, then the iterations of do loop in Newton’s method continuously to find the $\lambda_{mn}$ solution until the error less than 1 × 10^−6^.
